# Compatibility of fluazaindolizine and oxamyl with *Pasteuria penetrans* on spore attachment to juveniles of *Meloidogyne javanica* and *M. incognita*

**DOI:** 10.21307/jofnem-2020-070

**Published:** 2020-07-21

**Authors:** Eleni Nasiou, Tim Thoden, Iro V. Pardavella, Emmanuel A. Tzortzakakis

**Affiliations:** 1 Department of Viticulture, Vegetable Crops, Floriculture and Plant Protection, Institute of Olive Tree, Subtropical Crops and Viticulture, NAGREF, Hellenic Agricultural Organization-DEMETER, 32 Kastorias Street, Mesa Katsabas, 71307, Heraklion, Crete, Greece; 2 Corteva Agriscience™, Truderinger Str. 15, 81677 München, Germany

**Keywords:** Biological control, Compatibility, Integrated nematode management, Nematicides

## Abstract

Fluazaindolizine is a novel sulfonamide nematicide that is the active ingredient (a.i.) of Salibro™, a.i. Reklemel™. Its compatibility with *Pasteuria penetrans*, a bacterial parasite of root-knot nematodes (*Meloidogyne* spp.), was investigated in populations of *M. javanica* and *M. incognita*. Spores of a single *P. penetrans* isolate (*Pp* 3) or a blend of six isolates were incubated in the suspensions of fluazaindolizine (Salibro^TM^ 500SC, at 5, 50, and 250 ppm a.i.) and oxamyl (Vydate™ 10 L, 10% (a.i.) at 25 and 50 ppm a.i.) for 1, 7, and 21 days; controls were incubated in water. Thereafter, the suspensions were washed through a cellulose filter (3 µm) so as to remove the nematicide, and the spores retained on the filter were suspended in water. Juveniles (J2) were exposed in these spore suspensions in Petri dishes and the number of attached spores was recorded. Neither fluazaindolizine nor oxamyl, at all the tested dosages, had any negative effect on the rate of spore attachment. The spore encumbered J2 from some experiments were used to infect tomatoes. Females without egg masses were extracted from the roots after 50 days and checked for eggs in ovaries and mature spores of *P. penetrans*. Despite no mature spores present in the females, there was evidence of a low percentage of infection in a few treatments. A possible explanation is that since the bacterium had been kept stored in the form of dried roots for a long period, its ability to infect nematodes was decreased.

Salibro™ is a novel sulfonamide nematicide containing the active ingredient (a.i.) fluazaindolizine (Reklemel™ active). Its biochemical mode of action is presently unknown but in laboratory studies it caused adverse effects at concentrations around 1 to 50 ppm (a.i.) on various fitness parameters (motility, mobility, and infectivity) of *Meloidogyne incognita*, *M. javanica*, and *M*. *hapla* (Thoden and Wiles, 2019; Thoden et al., 2019).

*Pasteuria penetrans* is a mycelial and endospore forming bacterium parasitizing root-knot nematodes (RKN, *Meloidogyne* spp.) (Sayre and Starr, 1985), with potential as a biocontrol agent of these nematodes (Chen and Dickson, 1998). The first step in the *P. penetrans*-*Meloidogyne* interaction is the attachment of the bacterial spores on the cuticle of juveniles (J2). Spores are immobile and attachment takes place when the J2 moves and comes into contact with them. If a high number of spores attaches to the J2, movement is hindered and the nematode may not be able to enter the root. When a J2 with attached spores invades the root and starts feeding, the spores germinate and proliferate inside its body. The parasite is highly selective within the nematode causing minimum disturbance to the physiological functions of feeding, molting, and growth while selectively destroying the reproductive capacity. Finally, the adult female does not lay eggs but instead becomes filled with spores of the parasite (Chen and Dickson, 1998).

Modern nematicides should be compatible with biological control agents to be successfully applied in integrated control. Fluazaindolizine has shown low toxicity to the bacteriophagous nematode *Acrobeles buetschii* (Thoden and Wiles, 2019) and to many other beneficial nematodes (Thoden et al., unpubl. data). Previous studies have indicated the compatibility of *P. penetrans* with non-fumigant nematicides (Mankau and Prasad, 1972; Brown and Nordmeyder, 1985; Nishizawa, 1989; Tzortzakakis and Gowen, 1994). Therefore, the aim of the current work was to investigate whether pre-exposure of *P. penetrans* spores to two nematicides (fluazaindolizine and oxamyl) would affect both (i) the subsequent in vitro spore attachment to J2 as well as (ii) affect the infection of nematodes by the bacterium.

## Materials and methods

### Spore attachment to juveniles after pre-exposure to nematicides

The experiments evaluated different rates of the nematicides and different incubation times, and conducted in two years (2018-2019). The *P. penetrans* used were the single isolate *Pp* 3 from South Africa (V. Spaull) and a blend of six isolates: *Pp* 1 from Australia (G. Stirling), *Pp* 2 from the USA (R. Sayre), *Pp* 3, *Pp* PNG from Papua New Guinea (J. Bridge), *Pp* M from Malawi (A. Daudi), and *Pp* IC from the Ivory Coast (S. Gowen). All the isolates were obtained from the University of Reading, UK (Tzortzakakis et al., 1997), where they had been multiplied on various populations of *M. javanica* and *M. incognita* (Stirling and Wachtel, 1980) for several years. The stock material was in the form of dried roots containing *Meloidogyne* females filled with *P. penetrans* spores. This had been further multiplied in mixed populations of *M. javanica*/*M. incognita* from Crete over time and was stored at room temperature in the form of dried roots since 1995. To account for any variability in the pathogenicity of an individual single isolate of *P. penetrans*, in most experiments, we used the blend of isolates (Channer and Gowen, 1992; Tzortzakakis et al., 1997). The aqueous spore suspensions of spores were prepared by grinding roots with a pestle and mortar, suspending the material in distilled water, and sieving it through a 38 µm sieve to remove coarse root material. The spore density was estimated with a haemocytometer. The spore suspension was stored in a glass flask at 5^o^C for more than one month before being used in experiments to ensure sufficient hydration of spores (Tzortzakakis, 1993). A quantity of spores was taken from this suspension and incubated in plastic tubes at room temperature containing either fluazaindolizine (Salibro^TM^ 500SC, provided by Corteva Agriscience^TM^) at 5, 50, and 250 ppm (a.i.) or oxamyl (Vydate^TM^ 10 L, provided by Corteva Agriscience^TM^) at 25 and 50 ppm (a.i) for 1, 7, or 21 days, respectively. A plastic tube, containing the spore suspension and water instead of nematicides, served as control. Following incubation, the spores were filtered through a 3-µm cellulose filter using a syringe and then washed 3 to 5 times on the same filter to remove any adhering nematicide residues. The filter containing the *P. penetrans* spores was suspended in water and its surface scratched with a scalpel to remove the spores. This suspension containing the filter was then vortexed (2500 rpm for 1 min) to further remove spores remaining on the filter and homogenize the suspension before estimating the final spore density. The control for each experiment was spores incubated in water. In Experiments 4 to 6, the number of spores recovered after filtering varied considerably from that in the unfiltered suspension and therefore the number of spores per dish was different for each incubation period.

To test if the pre-treated spores attach to J2, freshly hatched J2 (0-4 days old) were obtained from eggs incubated in hatch dishes (Hussey and Barker, 1973). The J2 were transferred to either 5.5 cm (Experiment 1-3) or 3.5 cm (Experiment 4-6) diameter Petri dishes containing suspensions of 6,000 to 20,000 spores (with a final volume being 8 or 2-3 ml, respectively). The control (water-incubated spores) was checked after 24 h and if the number of attached spores was low, the incubation was extended for further 24 h. Therefore, after 24 or 48 h of incubation, in the spore suspension at 25 to 28^o^C, the number of spores that had attached to the cuticle of 10 randomly selected J2 was examined with an inverted microscope at 200 ×. Each treatment was replicated five times. Populations of either *M. javanica* or *M. incognita* were used for the experiments. The number of J2 per dish for each incubation period varied depending on the number of J2 harvested from the hatching dish. The nematode populations originated from Greece and were cultured on tomato plants in a growth room. Experiments 1 to 3 were conducted in 2018, while the Experiments 4 to 6 were done in 2019. The *Pp* blend used in 2018 was slightly different to that used in 2019. Both originated from the same stock material but had been multiplied in different mixed populations of *M. javanica*/*M. incognita* from Crete.

For Experiments 1 to 3, the dates of incubating the spore suspension in nematicides were arranged so that exposure of J2 in the attachment test was conducted the same day for all incubation periods within each nematode species. Thus there was a single control only, which was spores incubated in water for 21 days. The comparisons were between the single control and all the treatments (nematicides and incubation periods).

For Experiments 4 to 6, the comparisons of treatments were conducted per incubation period of the spores in the nematicide, with separate controls for each, as there were differences in the number of spores and J2 per dish for the various incubation periods.

### Infection of *Meloidogyne* spp. by *P. penetrans* spores previously exposed to nematicides

The J2 of *M. javanica* and *M. incognita* encumbered with spores of the *Pp* blend from Experiment 2 (1, 7, and 21 days incubation) were used to inoculate susceptible tomato plants (cv ACE) grown in 250 ml plastic cups filled with a commercial soil substrate (Humin Substrat, Klasmann-Deilmann GmbH, Germany). The content of each single Petri dish containing the spore encumbered J2 and free spores was inoculated into two holes around the plant stem that afterwards were covered with soil and irrigated lightly. For each experiment, there was an additional control, which consisted of J2 incubated in water without spores of *P. penetrans* for the same duration as those in the spore attachment test. Plants were kept in a glasshouse without artificially heating or lighting for 50 days (10-40^o^C) from middle September until beginning of November. The J2 from Experiments 4 to 6 were also added to pots containing tomato. In contrast to the first experiment, these plants were kept in a growth room at 25 to 32^o^C and 14 h photoperiod for 50 days. Afterwards, all plants were uprooted, the roots washed thoroughly, weighed, and the number of egg masses per root system assessed microscopically to estimate the number of egg masses per gram of root. Females without egg masses (10-15 per replicate) were randomly selected and extracted from the root tissue under a dissecting microscope. Those females were put in a drop of water, crushed with a cover slip to examine at 400× for the presence of oocytes, eggs, and mature spores of *P. penetrans*.

All results were analyzed with single ANOVA and treatment means (nematicides, untreated control with *P. penetrans*, and control without *P. penetrans* for pot tests) compared using LSD test at 5% level of significance. The analysis was conducted with the SAS University Edition.

## Results

### Spore attachment to juveniles after pre-exposure to nematicides

Results of Experiments 1 to 6, presented in Tables 1 and 2, indicate that for all nematicide treatments and at all incubation periods, the pre-treated spores attached to the J2 at numbers equal to or significantly higher (*p* ≤ 0.05) than the untreated spores (kept in water). Overall, spore attachment per J2 was highly variable and ranged from 0.32 to 15.50 for the control, 0.98 to 17.96 for those incubated in fluazaindolizine, and 0.94 to 8.04 for those incubated in oxamyl.

**Table 1.** Average number of spores per juvenile of *Meloidogyne javanica* (*M.j.*) and *M. incognita* (*M.i.*) exposed to spores of *Pasteuria penetrans* (*Pp* blend) which had been previously incubated for 1, 7, and 21 days in the nematicides fluazaindolizine and oxamyl.

	Experiment 1		Experiment 2		Experiment 3
Treatments	*M.j.*	*M.i.*	*M.j.*	*M.i.*	*M.j.*
Control (*Pp* untreated)	3.88 bc	6.22 cd	3.42 e	1.86 cd	15.50 ab
*Fluazaindolizine 5 ppm*					
1 day	3.46 c	8.72 ab	6.04 cde	1.14 cd	14.60 abc
7 days	5.20 abc	8.72 ab	7.56 bcd	1.86 cd	17.92 a
21 days	4.26 bc	7.52 bcd	12.70 a	1.90 cd	15.92 ab
*Fluazaindolizine 50 ppm*					
1 day	7.06 a	10.04 a	3.68 e	1.02 d	17.96 a
7 days	4.90 bc	7.72 bcd	6.58 bcde	1.68 cd	11.70 c
21 days	5.74 ab	7.88 bcd	9.76 abc	3.64 b	16.22 ab
*Fluazaindolizine 250 ppm*					
1 day					15.32 abc
7 days					14.66 abc
21 days					13.64 bc
*Oxamyl 25 ppm*					
1 day	4.94 bc	6.16 d	3.72 e	1.40 cd	
7 days	4.18 bc	6.40 cd	4.78 de	2.32 c	
21 days	5.42 abc	8.04 bc	10.04 ab	6.14 a	
Spores/dish (× 10^3^)	20	20	20	20	15
J2/dish	180	180	300	550	100
Incubation period	24 h	24 h	48 h	48 h	24 h

**Notes:** Each mean is the average of five replicates; means within columns followed by the same letter are not significantly different according to LSD test (*p* > 0.05); the control was spores incubated for 21 days in water.

**Table 2.** Average number of spores per juvenile of *Meloidogyne incognita* and *M. javanica* exposed to spores of *Pasteuria penetrans* (*Pp*) which had been previously incubated for 1, 7, and 21 days in the nematicides fluazaindolizine and oxamyl.

	*Pp* 3			*Pp* blend		
	Incubation period					
Treatments	1 day	7 days	21 days	1 day	7 days	21 days
Experiment 4						
* M. incognita*						
* *Control (*Pp* untreated)	1.26 c	1.64 b	3.46 b	0.78 c	2.86 a	0.32 b
* *Fluazaindolizine 50* *ppm	2.84 b	1.50 b	3.36 b	0.98 bc	2.42 a	1.06 a
* *Fluazaindolizine 250* *ppm	4.28 a	2.48 a	4.08 b	1.28 ab	2.20 a	1.08 a
* *Oxamyl 50* *ppm	4.68 a	2.76 a	5.02 a	1.33 a	2.38 a	0.94 a
* *Spores/dish (× 10^3^)	20	20	20	10	15	20
* *J2/dish	320	550	550	1,000	170	1,150
* *Incubation period	24 h	24 h	24 h	48 h	48 h	48 h
Experiment 5						
* M. javanica*						
* *Control (*Pp* untreated)			4.54 b			
* *Fluazaindolizine 50* *ppm			5.28 b			
* *Fluazaindolizine 250* *ppm			4.66 b			
* *Oxamyl 50* *ppm			7.70 a			
* *Spores/dish (× 10^3^)			15			
* *J2/dish			550			
* *Incubation period			24 h			
Experiment 6						
* M. javanica*						
* *Control (*Pp* untreated)	2.58 c	4.16 b	3.56 c	3.88 b	3.58 c	2.34 c
* *Fluazaindolizine 50* *ppm	3.60 b	3.96 b	4.38 b	4.02 b	4.82 b	2.96 b
* *Fluazaindolizine 250* *ppm	4.02 ab	3.78 b	4.74 b	4.10 b	5.16 ab	3.00 b
* *Oxamyl 50* *ppm	4.30 a	4.80 a	5.42 a	5.90 a	5.86 a	4.46 a
* *Spores/dish (× 10^3^)	20	20	20	10	6	8
* *J2/dish	1,000	1,000	700	350	300	400
* *Incubation period	48 h	48 h	48 h	48 h	48 h	48 h

**Notes:** Each mean is the average of five replicates; means within columns followed by the same letter are not significantly different according to LSD test (*p* > 0.05).

### Infection of *Meloidogyne* spp. by *P. penetrans* spores previously exposed to nematicides

The effect of nematicide treatment on spore infection of nematodes was tested using the spore-encumbered J2 from the attachment tests (Experiment 2 from Table 1 and all Experiments from Table 2). An additional control of J2 without spores was included in all evaluations. There was no difference in egg masses per gram root between the J2 with untreated spores and those without spores for Experiment 2 using the *Pp* blend (data not shown) indicating that this blend was ineffective in reducing root penetration by J2 and egg production by female nematodes under our greenhouse conditions. For Experiments 4 to 6, only Experiment 4 with *Pp*3 (seven-day incubation) showed fewer (*p* ≤ 0.05) egg masses per gram root for J2 with untreated spores compared to J2 without spores (Table 3); however, the evaluation of 21 days incubation showed numerically but not significantly fewer eggs masses for J2 with untreated spores than the J2 without spores. There was also no difference in egg masses per gram root between J2 without spores and any of the nematicide treatments for Experiment 4 (one-day incubation for *Pp* 3 and all incubation periods for *Pp* blend), Experiment 5, and Experiment 6 (one-day incubation for *Pp*3 and all incubation periods for *Pp* blend). Only data showing significant differences between the control of J2 without spores and J2 with nematicide-treated spores are shown in Table 3. In Experiment 4 and 6 with *Pp* 3, there was a tendency for some of the fluazaindolizine and oxamyl treated spores to be more effective in reducing (*p* ≤ 0.05) the number of egg masses than the untreated spores compared to the control without spores (Table 3). In four out of these six cases, the J2 had a higher number of attached spores before plant inoculation compared to the case of J2 with the untreated spores (Table 2). In experiments where the number of egg masses per gram root was significantly lower than the control without spores in some treatments, 10 to 15 females without egg masses per replicate from all treatments were checked for the presence of oocytes and eggs in ovaries and mature spores of *P. penetrans*. All the females from the controls without spores had either oocytes or eggs. A few females (< 10%) from treatments where spores were either exposed or not to nematicides were found without oocytes or eggs in their ovaries but mature spores were not found.

**Table 3.** Number of egg masses per gram of root of tomato plants infected with juveniles of *Meloidogyne incognita* and *M. javanica* pre-exposed to spores of *Pasteuria penetrans* (*Pp* 3).

	*Pp* 3	
	Incubation period	
Treatments	7 days	21 days
Experiment 4		
* M. incognita*		
* *Control without *Pp*	55.50 a	49.60 a
* Pp* untreated	10.50 b	42.60 ab
* *Fluazaindolizine 50 ppm	19.90 b	19.10 c
* *Fluazaindolizine 250 ppm	3.50 b	31.40 abc
* *Oxamyl 50 ppm	20.70 b	24.90 bc
Experiment 6		
* M. javanica*		
* *Control without *Pp*	103.43 a	86.89 a
* Pp* untreated	62.70 ab	72.55 ab
* *Fluazaindolizine 50 ppm	59.40 ab	57.31 bc
* *Fluazaindolizine 250 ppm	47.29 b	48.23 c
* *Oxamyl 50 ppm	79.56 ab	59.65 bc

**Notes:** Each mean is the average of five replicates; means within columns followed by the same letter are not significantly different according to LSD test (*p* > 0.05); the labels Experiment 4 and 6 indicate that J2 encumbered with spores from the respective in attachment experiments presented in Table 2 were used for plant inoculation.

## Discussion

In previously published studies, the compatibility of *P. penetrans* with organophosphate and carbamate nematicides had been proved in natural soil samples containing spores of the bacterium by applying nematicides and assessing the attachment of spores to nematodes with a bioassay (Mankau and Prasad, 1972; Nishizawa, 1989). In a pot test, combinations of *Pp* with the nematicides carbofuran and aldicarb synergistically reduced root galling (Brown and Nordmeyder, 1985), while in greenhouse studies in Crete, applications of oxamyl had an additive effect on the efficacy of *Pp* for controlling *Meloidogyne* spp. in tomato and cucumber (Tzortzakakis and Gowen, 1994). In all these studies, the spores of *P. penetrans* as well as the nematodes were exposed simultaneously to the nematicides. The fast acting nematicides that were used in those studies would have affected mobility of J2 (Hague and Gowen, 1987) and, therefore, spore acquisition. Thus, increasing mobility of J2 favors positively spore attachment while decreasing mobility has a negative effect. The nematicides at low non-lethal dosages in treated soil might have increased movement of nematodes and the probability of J2 contacting more spores (Brown and Nordmeyder, 1985). Therefore, in these previous investigations, the direct effect of nematicides on spore viability cannot be separated from positive or negative effects on nematode mobility and spore acquisition.

In this research, we incubated the spores in nematicides and washed them afterwards so as to minimize the effect of nematicides on J2 mobility. Overall, the experiments indicated that both fluazaindolizine and oxamyl are compatible with *P. penetrans* with regard to spore attachment and therefore might be successfully applied in integrated control of RKN.

Fluctuating soil temperature may delay the development of *P. penetrans* inside the females (Hatz and Dickson, 1992; Giannakou et al., 1999; Darban et al., 2005). It is possible that delayed development of *P. penetrans* allowed egg production by infected females. This may explain the absence of reduction in egg masses per gram of root for Experiment 2 in the greenhouse without temperature regulation. However, the temperature in the growth room for Experiments 4 to 6 was more stable and favorable for infection and development by *P. penetrans* within a 50-day period (Stirling, 1981; Darban et al., 2005). In a few treatments, there was evidence for infection by *P. penetrans* as indicated by lower number of egg masses and few signs of egg production within females regardless of whether spores had been treated with nematicide or not. However, no mature spores of *P. penetrans* were observed within females without egg masses in all cases. Since we did not have access to a commercial product of *P. penetrans*, we used the only available material which was *P. penetrans* in the form of dried roots kept stored for 23 years. Reduced infection by spores stored in the form of dried roots for long periods (6 or 11 years) has been previously reported (Espanol et al., 1997; Giannakou et al., 1997). In the current study, spores attached readily to J2 but their ability to germinate and infect may have been diminished, considering that these isolates were infectious when were freshly produced, 23 years ago. Furthermore, the life cycle of the bacterium may have been prolonged due to this long storage, resulting in no mature spores in females 50 days after plant inoculation. Although there is substantial evidence that the incubation of spores in fluazaindolizine and oxamyl do not affect their ability to attach to J2, the effect of these nematicides on spore infection was not clearly established. In this study the long storage of *P. penetrans* in the form of dried roots did not have any important effect on spore attachment but probably reduced their ability to infect.
